# The heterogeneity in GABA_A_ receptor-mediated IPSC kinetics reflects heterogeneity of subunit composition among inhibitory and excitatory interneurons in spinal lamina II

**DOI:** 10.3389/fncel.2014.00424

**Published:** 2014-12-11

**Authors:** Charalampos Labrakakis, Uwe Rudolph, Yves De Koninck

**Affiliations:** ^1^Unité de Neurosciences Cellulaires et Moléculaire, Institut Universitaire en Santé Mentale de QuébecQuébec, QC, Canada; ^2^Department of Biological Applications and Technology, University of IoanninaIoannina, Greece; ^3^Laboratory of Genetic Neuropharmacology, McLean Hospital, and Department of Psychiatry, Harvard Medical SchoolBelmont, MA, USA; ^4^Department of Psychiatry and Neuroscience, Université LavalQuébec, QC, Canada

**Keywords:** GABA_A_ receptors, IPSCs, decay kinetics, spinal dorsal horn, subunit composition

## Abstract

GABAergic inhibition displays rich functional diversity throughout the CNS, which arises from variations in the nature of inputs, subunit composition, subcellular localization of receptors and synapse geometry, or reuptake mechanisms. In the spinal dorsal horn (SDH), GABA_A_ and glycine receptors play a major role in the control of excitability and accuracy of nociceptive processing. Identifying which components shape the properties of the inhibitory synapses in different cell types is necessary to understand how nociceptive information is integrated. To address this, we used transgenic mice where inhibitory interneurons express GAD65-EGFP. We found that GABA_A_, but not glycine receptor-mediated evoked IPSCs displayed slower kinetics in EGFP+ vs. EGFP− interneurons. GABA_A_ miniature IPSC decay kinetics showed a large variability in both populations, however the distribution of decays differed between EGFP+ and EGFP− interneurons. The range of mIPSC decay kinetics observed was replicated in experiments using rapid application of GABA on outside-out patches taken from SDH neurons in slices. Furthermore, GABA_A_ decay kinetics were not affected by uptake blockers and were not different in mice lacking δ or α5 subunits, indicating that intrinsic channel properties likely underlie the heterogeneity. To identify whether other α subunits shape the various kinetic properties observed we took advantage of knock-in mice carrying point mutations in either the α1, α2, or α3 subunits rendering Ro 15-4513 a selective agonist at the benzodiazepine modulatory site. We found that α1 and α2 subunit underlie the fast decaying component of IPSCs while the slow component is determined by the α3 subunit. The differential distribution of GABA_A_ subunits at inhibitory synapses thus sculpts the heterogeneity of the SDH inhibitory circuitry. This diversity of inhibitory elements can be harnessed to selectively modulate different components of the spinal nociceptive circuitry for therapeutic interventions.

## Introduction

The spinal dorsal horn (SDH) serves both as a relay and processing station for somatic sensory information entering from the peripheral sensors in transit to the higher brain centers. Primary afferents carrying somatic sensory input terminate in the spinal cord in a laminar distribution pattern. Aδ - and peptidergic C-fibers carry nociceptive, thermal, itch and innocuous tactile information and terminate in lamina I and outer II while non-peptidergic C-fibers terminate predominantly in inner lamina II (Ribeiro-da-Silva and De Koninck, 2008). Incoming nociceptive information converges onto projection neurons in lamina I following processing through a local network of interneurons in lamina II that control the final output. The inhibitory neurotransmitters GABA and glycine and their receptors in the dorsal horn play multiple roles in the control of information flow, the discrimination of sensory modalities and thus in securing the accuracy in the transmission of sensory information. Indeed, intrathecal GABA_A_ receptor agonists increase the nociceptive threshold in rats (Hammond and Drower, 1984), while administration of GABA_A_ or glycine receptor antagonists produce pain hypersensitivity and allodynia (Yaksh, 1989; Sivilotti and Woolf, 1994; Sorkin and Puig, 1996). Recent work suggests that reduced inhibition in the dorsal horn underlies neuropathic and inflammatory pain (Coull et al., 2003; Harvey et al., 2004). Such a disinhibition lowers the control of interconnected sensory networks allowing unorthodox information flow from normally innocuous inputs to nociceptive pathways (Baba et al., 2003; Torsney and MacDermott, 2006; Keller et al., 2007).

GABAergic transmission displays a high level of heterogeneity in the brain. Several types of interneurons that release GABA form synapses with different properties on various compartments of the target cells. An important potential substrate of heterogeneity at GABA_A_ synapses is their molecular composition: 19 subunits are available to assemble the pentameric GABA_A_ receptors (Olsen and Sieghart, 2009). Several subunits, including α1, α2, α3, and α5, are expressed in the SDH (Bohlhalter et al., 1996). Additionally, several distinct morphological and histochemical classes of interneurons that can realease GABA have been identified (Laing et al., 1994). However, despite the important functions fulfilled by inhibitory interneurons, little is known on the functional organization of inhibitory networks, their connections, mode of operation or properties. Such knowledge is imperative in light of recent work unveiling new opportunities for the development of selective treatments for chronic pain by targeting GABA_A_ receptors (Knabl et al., 2008; Zeilhofer et al., 2009). However, as we asserted previously, inhibitory interneurons in the SDH can play a dual role in moderating spinal excitability, restraining it by feed forward inhibition of the polysynaptic excitatory relay circuit, but also promote it by reciprocal silencing of other inhibitory interneurons (Labrakakis et al., 2009). Hence, the complex functionality of inhibitory networks has to be taken into account (Cossart et al., 2005). In this context, we explored the properties of GABA_A_–mediated synaptic events in subclasses of interneurons in the SDH, using mice that express EGFP under the control of the inhibitory neuron promoter glutamate decarboxylase (GAD65). We found that the properties of GABA_A_ IPSCs are different between inhibitory and presumably excitatory interneurons. In addition we show that the heterogeneity of kinetic properties we observed results from difference in subunit composition of the receptors. These findings have bearings on strategies for selective modulation of subcomponents of the inhibitory circuitry in the SDH.

## Materials and methods

### Ethical approval

All experiments were performed in accordance with regulations of the Canadian Council on Animal Care. Experimental procedures were approved by the Comité de protection des animaux de l'Université Laval.

### Animals

Several genetically modified mouse lines were used in this study. Heterozygous GAD65-EGFP transgenic mice express the enhanced green fluorescent protein (EGFP) under the control of the GAD65 promoter (Lopez-Bendito et al., 2004; Labrakakis et al., 2009). Gabra5−/− and Gabrd−/− mice are deficient in the GABA_A_ receptor α5 and δ subunits respectively (Mihalek et al., 1999; Collinson et al., 2002). Homozygous α1(H101R), α2(H101R), and α3(H126R) mice are carrying a mutation substituting a histidine for an arginine at the benzodiazepine binding site of the GABA_A_ receptor α1, α2 and α3 subunits, respectively (Rudolph et al., 1999; Low et al., 2000). Wild type and genetically modified mice were all of the C57BL/6J, C57BL/6J x129/SvEv (α5−/−) or C57BL/6J × 129/SvJ (δ −/− and δ +/+) background. GAD65-EGFP mouse phenotype was determined by the presence of GFP. The genotype of all other mouse lines was ascertained by PCR.

### Preparation of spinal cord slices

Adult mice (3–6 months) of both sexes, were anesthetized with ketamine/xylazine and perfused intracardially with ice-cold oxygenated (95% O_2_, 5% CO_2_) sucrose substituted ACSF containing (mM) 252 sucrose, 2.5 KCl, 1.5 CaCl_2_, 6 MgCl_2_, 10 glucose, 26 NaHCO_3_, 1.25 NaH_2_PO_4_ and 5 kynurenic acid as previously described (Chery et al., 2000; Labrakakis et al., 2009). Mice were decapitated, the spinal cord was removed by hydraulic extrusion and 250 μm thick parasagittal slices were cut from the lumbar portion. Slices were transferred in normal oxygenated ACSF (126 NaCl, 2.5 KCl, 2 CaCl_2_, 2 MgCl_2_, 10 glucose, 26 NaHCO_3_, 1.25 NaH_2_PO_4_) and incubated at 33°C for 1 h and then kept at room temperature until recording.

### Electrophysiology

Slices were transferred in the recording chamber and continuously superfused at 2–3 ml/min with oxygenated ACSF at room temperature (23–26°C). Dorsal horn neurons were visualized with a Zeiss Axioplan2 microscope equipped with infrared “gradient-contrast” optics, epifluorescence and a × 40 water immersion objective. Patch pipettes (borosilicate glass; 6–8 MΩ) were filled with (in mM) 135 CsCl, 10 HEPES, 2 MgCl_2_, 0.5 EGTA. For some recordings, CsCl was substituted by 130 CsSO_3_CH_3_/5 CsCl. Whole cell patch clamp recordings were made using a Multiclamp 700B amplifier (Molecular Devices, Sunnyvale, CA). Access resistance was monitored periodically throughout the experiment. Recordings were not analyzed if access resistance was unstable or exceeded 30 MΩ. Data were low-pass filtered at 3 kHz, digitized at 10 kHz and acquired with the Strathclyde electrophysiology software (WinWCP and WinEDR courtesy of Dr. J. Dempster, University of Strathclyde, Glasgow, UK). All recordings in this study are from neurons with their somata located in laminae II.

Monosynaptic IPSCs were evoked focally by electrical stimulation (30–70 μ A, 250 μs) via patch pipette filled with ACSF and placed 50–100 μm from the soma of the recorded cell as described previously (Labrakakis et al., 2009). Single stimuli were delivered every 10 s. The mIPSCs were detected and analyzed using Mini Analysis (Synaptosoft, Decatur, GA) and locally designed software (YDK). Detection thresholds for mIPSCs were set at three times the RMS of noise. Decay time constants were fitted using automated least square algorithms. The necessity to introduce additional exponential components to the fits was first judged on the basis of visual inspection. When the merit of additional components was not obvious, further statistical analysis was applied as previously described (Chery and De Koninck, 1999). The weighted decay time constant (τ_w_) was calculated from dual-exponential fits using the following equation: τ_w_ = (τ_1_A_1_ + τ_2_A_2_)/(A_1_ + A_2_) where τ_1_ and τ_2_ are the fast and slow decay time constants and A_1_ and A_2_ are the equivalent amplitude weighting factors. The cumulative probability plots of mIPSC decay τ's were fitted by mixtures of Gaussian distributions:

$$P(x)=\sum_{i\;=\;1}^{n}\frac{R_{i}}{2}\left(1+erf\left(\frac{x_{i}-{\bar{x}}_{i}}{\sigma_{i}\sqrt{2}}\right)\right)$$

Where *R*_1_, …, *R*_*n*_, are the ratios of the *n* normal distributions (such that $\sum_{i\;=\;1}^{n}R_{i}=1$), *x*_*i*_, …, *x*_*n*_ are the means and σ_1_, …, σ_*n*_, the standard deviations (Cordero-Erausquin et al., 2009). The necessity to introduce additional Gaussian components to the fits was judged first on the basis of visual inspection of the fitted curves superimposed onto the data. When the merit of additional components was not obvious, an *F*-test was used to assess how the additional component improved the value of the reduced chi-square: *F*_*x*_ = Δχ^2^/χ^2^_ν_ (where χ^2^_ν_ = χ^2^/ν and the factor ν = *N* − *n* is the number of degrees of freedom left after fitting *N* data points to the *n* parameters; df_1_ = 3 and *df*_2_ = ν). The critical value for the merit of additional components was set at a low level (*p* < 0.0001) to favor parsimony of the fitted function (De Koninck and Mody, 1994; Chery and De Koninck, 1999). For the analysis of the effects of Ro 15-4513 (Sigma) on different populations of mIPSCs we categorized them as fast if their decay τ_w_ was <100 ms or slow if their decay τ_w_ was >100 ms. The 100 ms cut off was chosen on the basis of the Gaussian distributions fitted from the cumulative probability plots; 100 ms is the interface between the slower and the faster Gaussian components (**Figure 4A** right).

### Rapid agonist application on excised outside out patches

Stable outside out membrane patches were excised by pulling the pipette away from a whole-cell patched neuron. Excised patches were placed at the interface of a double-bore glass flow pipe with control ACSF and 1 mM GABA-containing solutions. Rapid exchange was achieved by fast displacement using a piezoelectric positioning system (Physik Instrumente, Germany) as previously described (Bowie et al., 1998). Solution exchange speed was determined at the end of each experiment by measuring open tip currents resulting from the liquid junction potentials between control and 0.5x ACSF (rise and decay typically ranged between 400 and 500 μs). Data were discarded from patches in which the liquid junction currents exhibited slow rise times.

### Simulation, analysis, and statistics

To simulate the effect of dendritic filtering on mIPSC decay kinetics, a simple ball and stick model was used in NEURON (Hines and Carnevale, 1997) software. Dendrite diameter was 1.5 μm, axial resistivity 300 Ω cm, membrane capacitance 1 μF/cm^2^. A passive leak conductance of 0.2 mS/cm^2^ was distributed throughout the cell. Voltage clamp series resistance was set to 30 MΩ. The synapses were modeled as a conductance change at different distances along the dendrite. For this a recorded mIPSC with fast decay kinetics (decay 27.3 ms, 10–90% rise: 1.2 ms) was used. This model is adequate to compare the relative effects of dendritic filtering on rise time vs. decay time but cannot accurately describe the effect of neuron morphology on dendritic filtering.

To model the effect of receptor binding affinity on the decay kinetics, simulated synaptic currents were generated with Channelab (Synaptosoft, Decatur, GA). The 5th-order Runga-Kutta numerical integrator was used for simulated macroscopic currents. For simulated mIPSCs, the Monte Carlo simulator was used. Gaussian noise (3 kHz) was added to the simulated mIPSCs. The latter were analyzed similarly to real mIPSCs, using locally developed software.

Normality of the data was tested with the Shapiro-Wilk test. Parametric data are expressed as mean ± s.e.m. and Student's independent *t*-test was used to analyze differences. One-Way ANOVA was used to analyze IPSC differences between wt and point mutated knock-in mice and *post-hoc* tests were obtained with Bonferroni or Tukey corrections to compare means. The non-parametric, distribution-free Kolmogorov–Smirnov test was used to compare cumulative probability distributions. Unless otherwise noted, n's represent number of neurons.

## Results

### Differential decay kinetics of evoked GABA_A_ IPSCs between lamina II interneuron subpopulations

**Figure 1:**
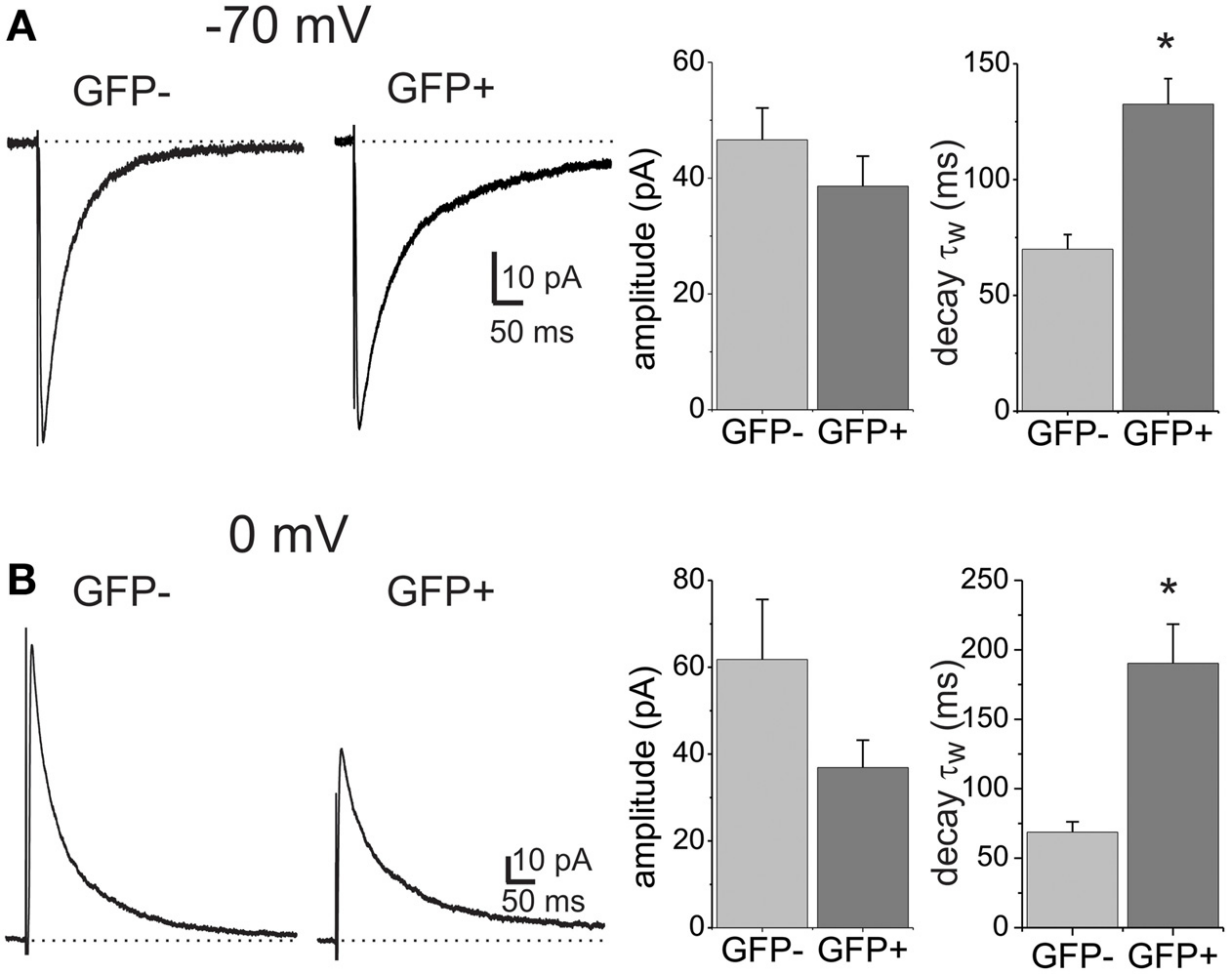
**GABA_A_ receptor-mediated eIPSCs differ in their decay kinetics between lamina II dorsal horn neuron subpopulations**. **(A)** IPSCs evoked by focal stimulation recorded from a EGFP negative (GFP−) and a EGFP positive (GFP+) neuron. Recordings were made at a −70 mV holding potential with CsCl containing pipettes. Histograms show the mean amplitude and weighted decay time constant (τ_w_) in the two cell populations. The asterisk denotes significant difference (*n* = 21 for GFP− and *n* = 28 for GFP+). **(B)** Example traces of eIPSCs recorded from GFP− and GFP+ cells at a holding potential of 0 mV and using CsSO_3_CH_3_-containing patch pipettes. Histograms show the mean amplitude and decay τ_w_. Asterisk denotes significant difference (*n* = 12 for GFP− and *n* = 14 for GFP+).

To investigate heterogeneity of GABAergic transmission in the dorsal horn we used parasagittal slices from GAD65-EGFP transgenic mice. Neurons were visually identified under epifluorescence as EGFP+ and thus inhibitory or as EGFP− and mainly excitatory interneurons (Lopez-Bendito et al., 2004; Labrakakis et al., 2009). Focally evoked monosynaptic GABA_A_ IPSCs were recorded in the presence of glutamate and glycine receptor blockers, CNQX (10 μM), APV (40 μM), and strychnine (0.5 μM). At a holding potential of −70 mV with high Cl^−^ pipettes, eIPSCs were inward and showed complex deactivation kinetics consisting of two exponentials both in EGFP− and EGFP+ neurons (Figure 1A). eIPSC amplitudes were similar in the two populations (46.6 ± 5.5 pA in EGFP− and 38.6 ± 5.2 pA EGFP+ neurons, *p* > 0.05, *n* = 21 and 28 respectively). Comparison of the decay kinetics, revealed differences in the weighted decay time constant (τ_w_; 69 ± 6.3 ms, *n* = 21 in EGFP− and 132.5 ± 11.1, *n* = 28 in EGFP+ neurons; *p* < 0.001). Decay kinetics in EGFP− or EGFP+ cells did not differ between male and female mice (65.8 ± 6.4 and 75.4 ± 12.3 for EGFP−, 150.7 ± 17.8 and 116.6 ± 12.9 for EGFP+, male and female respectively; ANOVA, *p* > 0.05). Additional experiments were performed using low Cl^−^ pipettes and a holding potential of 0 mV. Under these conditions the reversal potential for Cl^−^ and GABA_A_ currents is more negative than the holding potential. As expected, focal stimulation produced outward eIPSCs that displayed double exponential decay kinetics. In agreement with the previous experiments eIPSCs decay kinetics were significantly slower in EGFP+ neurons (68.7 ± 7.4 vs. 190.3 ± 28.2 ms for EGFP− and EGFP+ respectively, *n* = 12 and 14, *p* < 0.05) while amplitudes were not significantly different (61.8 ± 13.8 pA, *n* = 12 for EGFP− and 36.9 ± 6.3 pA, *n* = 14 for EGFP+, *p* > 0.05; Figure 1B). Finally, comparison of the biexponential decay components separately showed that the main difference between the two interneuron subpopulations was in the slow decay component in both sets of experiments (Table 1).

**Table 1:** **Summary of decay kinetic values for GABA_A_ eIPSCs recorded at holding potentials of −70 and 0 mV**.

	**V_**h**_: −70 mV holding**					**V_**h**_0 mV**				
	**A_**1**_**	**τ**_**1**_	**A_**2**_**	**τ**_**2**_	***n***	**A_**1**_**	**τ**_**1**_	**A_**2**_**	**τ**_**2**_	***N***
GFP−	31.5 ± 4.7	31.9 ± 1.8	15.1 ± 1.5	152.0 ± 19	21	45.4 ± 11.4	35 ± 3.3	16.4 ± 3.2	161 ± 21.1	12
GFP+	24.3 ± 3.3	43.0 ± 2.3^*^	14.3 ± 2.1	277.9 ± 21.4^**^	28	22.6 ± 3.9	45.5 ± 4.1	14.3 ± 2.7	398.2 ± 49^**^	14

* p < 0.05;

** p < 0.01; comparison between EGFP− and EGFP+ values.

### Decay kinetics of glycinergic eIPSCs were similar among interneuron subpopulations

**Figure 2:**
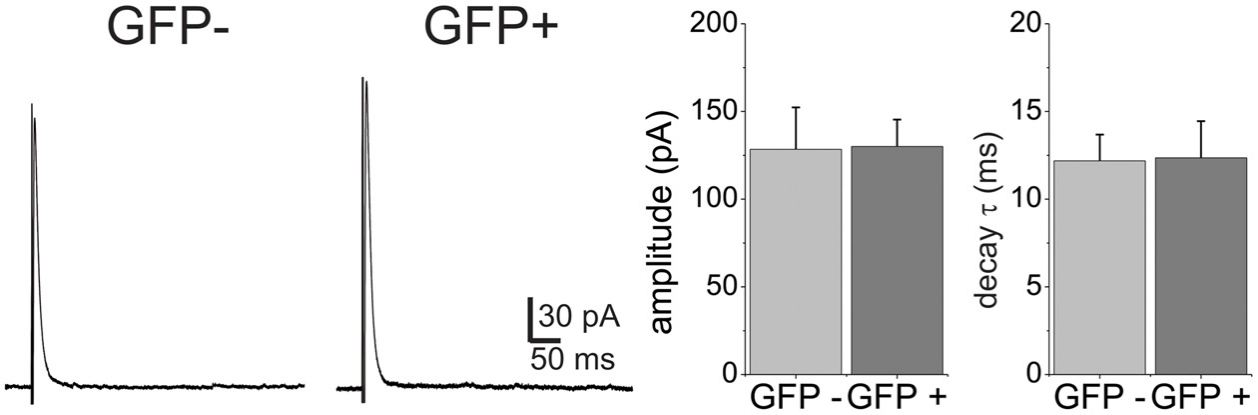
**Glycine receptor-mediated eIPSCs have similar decay kinetics in GFP+ and GFP− neurons**. Example traces of eIPSCs were recorded from GFP− and GFP+ neurons at a holding of 0 mV. Neither amplitude nor decay time constant were significantly different in the two populations (*n* = 6 for GFP− and *n* = 6 for GFP+).

In addition to GABA, fast inhibitory neurotransmission in the spinal cord is also carried by glycine. A large proportion of the GABAergic inhibitory interneurons in the lamina II of the dorsal horn also co-release glycine, while all the glycinergic neurons also contain GABA (Todd and Sullivan, 1990; Chery and De Koninck, 1999). We asked the question if glycine receptor mediated synaptic transmission also displays different decay properties between the EGFP+ and EGFP− neuron populations. Glycinergic eIPSCs were pharmacologically isolated in the presence of APV, CNQX and the GABA_A_ receptor antagonist SR95531 (10 μM) and recorded at 0 mV. Evoked eIPSCs under these conditions displayed monoexponential decay kinetics (Figure 2). Comparison of amplitudes (128.4 ± 23.9 pA vs. 130 ± 15.34, *p* > 0.05) and decay time constants (τ = 12.35 ± 2.1 vs. 12.2 ± 1.5) showed that they were not different in EGFP− (*n* = 8) and EGFP+ (*n* = 10) neurons. This indicates that decay kinetic differences are restricted to the GABA_A_ transmission and is not due to a difference in electrotonic properties of the cell populations.

### Differences in GABA_A_ mIPSCs between interneuron subpopulations

**Figure 3:**
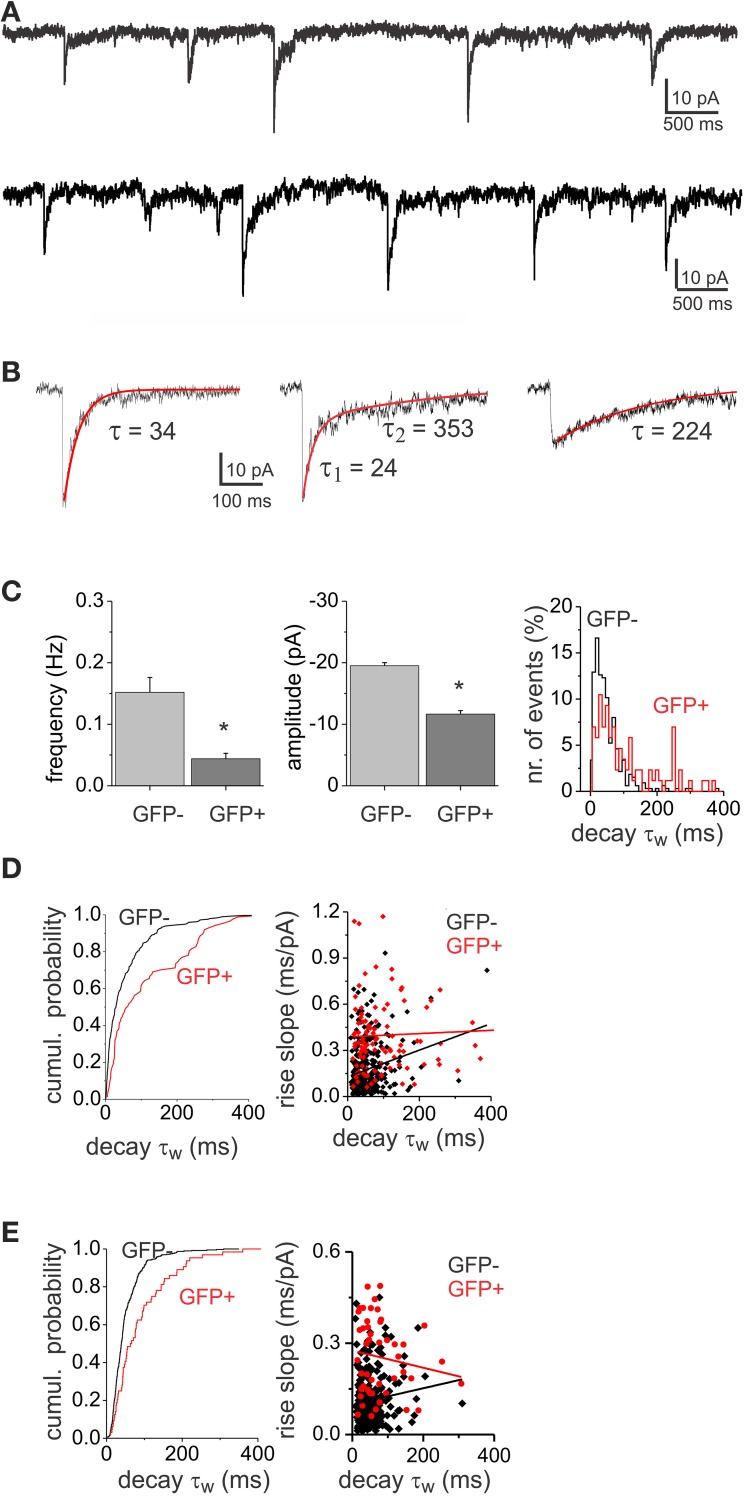
**GABA_A_ mIPSCs show both fast- and slow-decay kinetics. (A)** Example of a recording of mIPSCs from a GFP+ (top) and a GFP− (bottom) neuron displaying variable decay kinetics. **(B)** Different examples of single mIPSCs with variable decay kinetics: fast (left), mixed (middle) and slow decay (right) events. **(C)** Histograms of mIPSC frequency and amplitude showing significant differences (asterisk) between GFP− (*n* = 6) and GFP+ (*n* = 6) cell populations. The population distribution histogram of decay time constants (left, bin width: 20 ms) shows that GFP− and GFP+ display different proportions of fast and slow mIPSCs. **(D)** The cumulative probability plot (left) shows differential distribution of mIPSC decay τ_w_ between GFP− and GFP+ cell populations (left, *p* < 0.05 Kolmorgorov-Smirnov test). The plot consists of pooled mIPSC decay time constants from 6 GFP− and 6 GFP+ cells. The same number of consecutively occurring mIPSCs from each cell was used. The relationship between 10 and 90% rise slope and the decay τ_w_ in EGFP− and EGFP+ mIPSCs is plotted on the right. There was no correlation between mIPSC rise and decay neither in GFP− (*r* = 0.22) nor in GFP+ (*r* = 0.05) neurons. **(E)** Cumulative probability plot (left) and rise-slope/decay τ_w_ relationship plot (right) of the subset of data with rise times <4 ms. The distribution of decay τ_w_ between GFP− and GFP+ cells was different (*p* < 0.05), while there was no correlation between rise time and decay τ_w_ in GFP− (*r* = 0.11) and GFP+ (*r* = 0.14) neurons.

Evoked stimulation results in the synchronous recruitment of multiple release sites which may lead to accumulation of the neurotransmitter in the synaptic cleft that may spillover out of the synapse to extrasynaptic receptors (Isaacson et al., 1993). Alteration of the transmitter time course in the cleft or activation of distant receptors by long range diffusion could be responsible for the slow decay component in the eIPSCs and for the observed differences. To minimize the effect that diffusion or massive accumulation of neurotransmitter might have in shaping the decay kinetics of GABA_A_ IPSCs we looked at quantal release events in EGFP− and EGFP+ interneurons. Miniature IPSCs (mIPSCs) were recorded in the presence of TTX (1 μM) and strychnine (0.5 μM; Figures 3A,B) at a holding potential of −70 mV with high Cl^−^ pipettes. Mean mIPSC frequencies were higher in EGFP− interneurons (0.15 ± 0.02 Hz, *n* = 6) compared to EGFP+ interneurons (0.04 ± 0.01 Hz, *n* = 6, *p* < 0.05). In addition mIPSCs in EGFP− cells were found to have larger amplitudes (−19.54 ± 1.8 pA, *n* = 6) than EGFP+ cells (−11.57 ± 1.2 pA, *n* = 6, *p* < 0.05; Figure 3C). The mIPSCs showed a large variability in their decay kinetics (Figure 3C) in both cell populations. Figure 3D shows the cumulative probability plot of the τ_w_ showing differential distributions in the two interneuron populations. To reveal if the large variability of decay kinetics could be the result of electrotonic filtering we compared the decay τ_w_ to the rise slope (10–90 rise time/amplitude) of the mIPSCs. We found no correlation between rise and decay times (Figure 3D), indicating that the decay kinetics variability was not a result of filtering of mIPSC from remote synaptic locations. In addition, to limit the influence of presumably strongly filtered mIPSCs, we confined our analysis to a subset of mIPSCs with faster rise times possibly arising closer to the soma. Cumulative probability plots of the decay τ_w_ (Figure 3E) confirm the differential distribution of decays in the two interneuron populations, while no correlation could be found between rise time and decay τ_w_ for this subset of our data.

**Figure 4:**
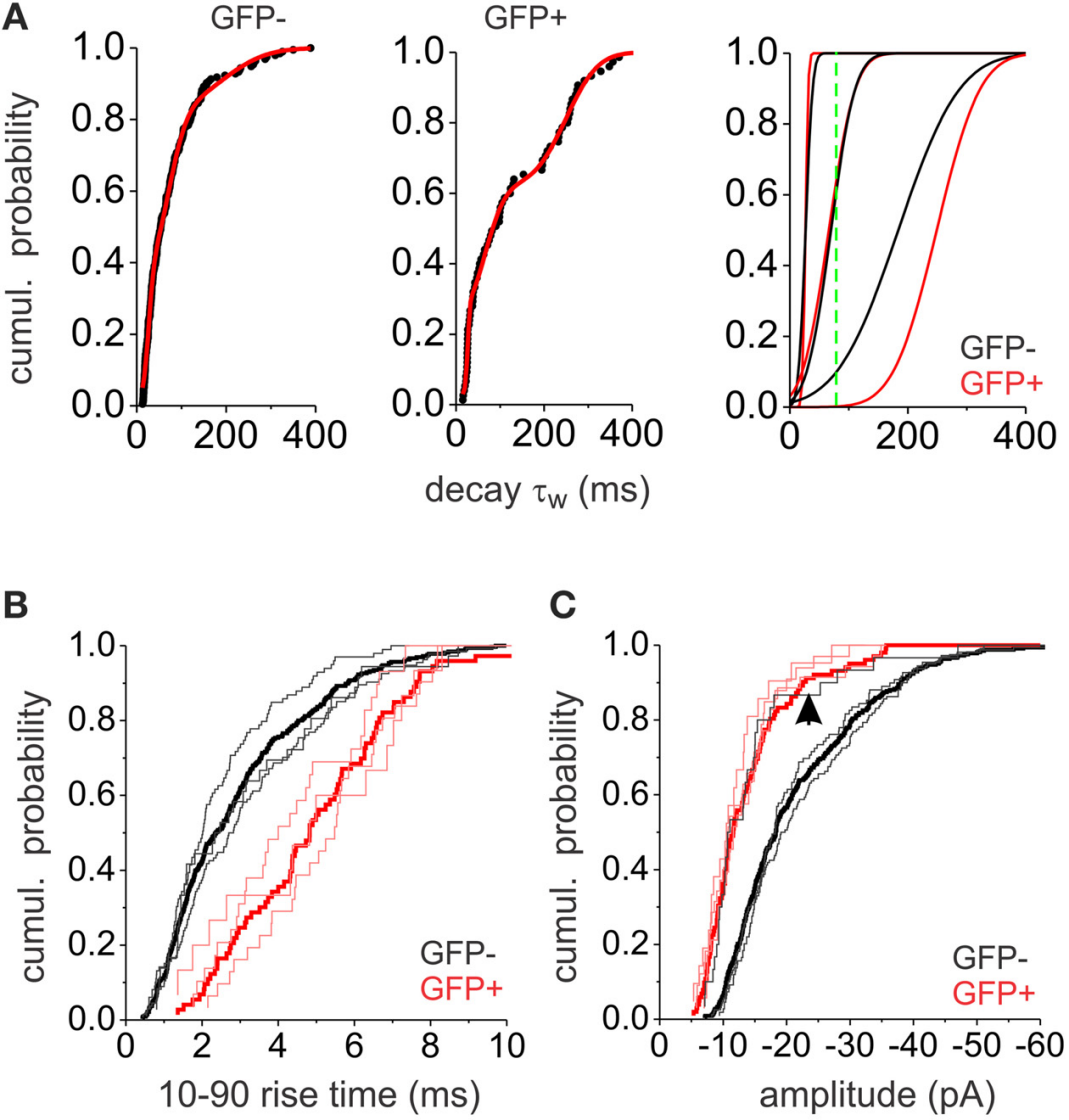
**Analysis of mIPSC populations**. **(A)** The cumulative probability plots for GFP− and GFP+ neurons (left and middle) were fitted with the sum of three normal distribution functions (red line). The resulting three distributions are plotted (right) separately (black lines for GFP−, red lines for GFP+. The green line is showing the 100 ms cut-off point used to separate slower from faster events). **(B)** Cumulative probability plots of the 10–90% rise times for GFP− (black) and GFP+ (red) mIPSCs. Thick lines show the total mIPSC population while the thin lines show subpopulations of mIPSCs based on the three Gaussian distributions distinguished in **(A)**. **(C)** Cumulative probability plots of the mIPSC amplitudes in GFP− (black) and GFP+ (red) interneurons. Thick lines show the total mIPSC population while the thin lines show subpopulations of mIPSCs based on the three Gaussian distributions distinguished in **(A)**. The arrow denotes the cumulative distribution of the amplitudes in the slow decaying subpopulation of GFP− interneurons. All plots consist of pooled data from 6 GFP+ and 6 GFP− neurons. The same number of consecutively occurring mIPSCs from each cell was used.

The above results indicate that electrotonic filtering is not the main factor shaping decay kinetics. We thus considered the possibility that the large decay variability was due to different mIPSC populations with variable properties. Indeed, the cumulative probability plots indicate multiple possible subpopulations of mIPSC decays. We were able to fit the data with a mixture of three Gaussian distributions (Figure 4A). The means and standard deviations (SD) of the three distributions were similar in both interneuron populations (Table 2). This indicates that the same subpopulations of mIPSCs make up the synaptic events of both EGFP− and EGFP+, however the relative contribution of each mIPSC subpopulation was different. To investigate the rise time and amplitude distributions within these mIPSCs subpopulations we divided the mIPSCs in three groups based on the means and SD derived from the Gaussian fits (mean ± 1 × SD). Rise time distribution for each group of mIPSCs was similar within each interneuron type and followed the distribution of the total population; however, mIPSCs in EGFP+ interneurons showed slower rise times than EGFP− mIPSCs (Figure 4B). This shows that rise time is cell type dependent and confirms that decay τ_w_ does not depend of the rise time. Amplitude distribution for the two faster decaying mIPSC groups was similar within each interneuron population with those in EGFP+ interneurons showing smaller size (Figure 4C). Interestingly, the amplitudes of the slower decaying mIPSCs in EGFP− interneurons show smaller size distributions akin to those in EGFP+ cells.

**Table 2:** **Decay τ means (μ), standard deviations (SD) and weighting factor results from the mIPSC population analysis**.

	**EGFP−**			**EGFP+**		
	**Weight**	**μ**	***SD***	**Weight**	**μ**	***SD***
1st	0.34	26.6	9.5	0.23	26.4	3.9
2nd	0.46	69.8	32.4	0.38	66.1	35.3
3rd	0.2	183.8	89.7	0.39	249.0	57.9

These results show that GABAergic synapses in SDH cells express a variety of decay kinetics and that the relative distribution of synapses with different kinetics is cell type dependent.

### Variations in eIPSCs decay kinetics are not due to differences in GABA reuptake

**Figure 5:**
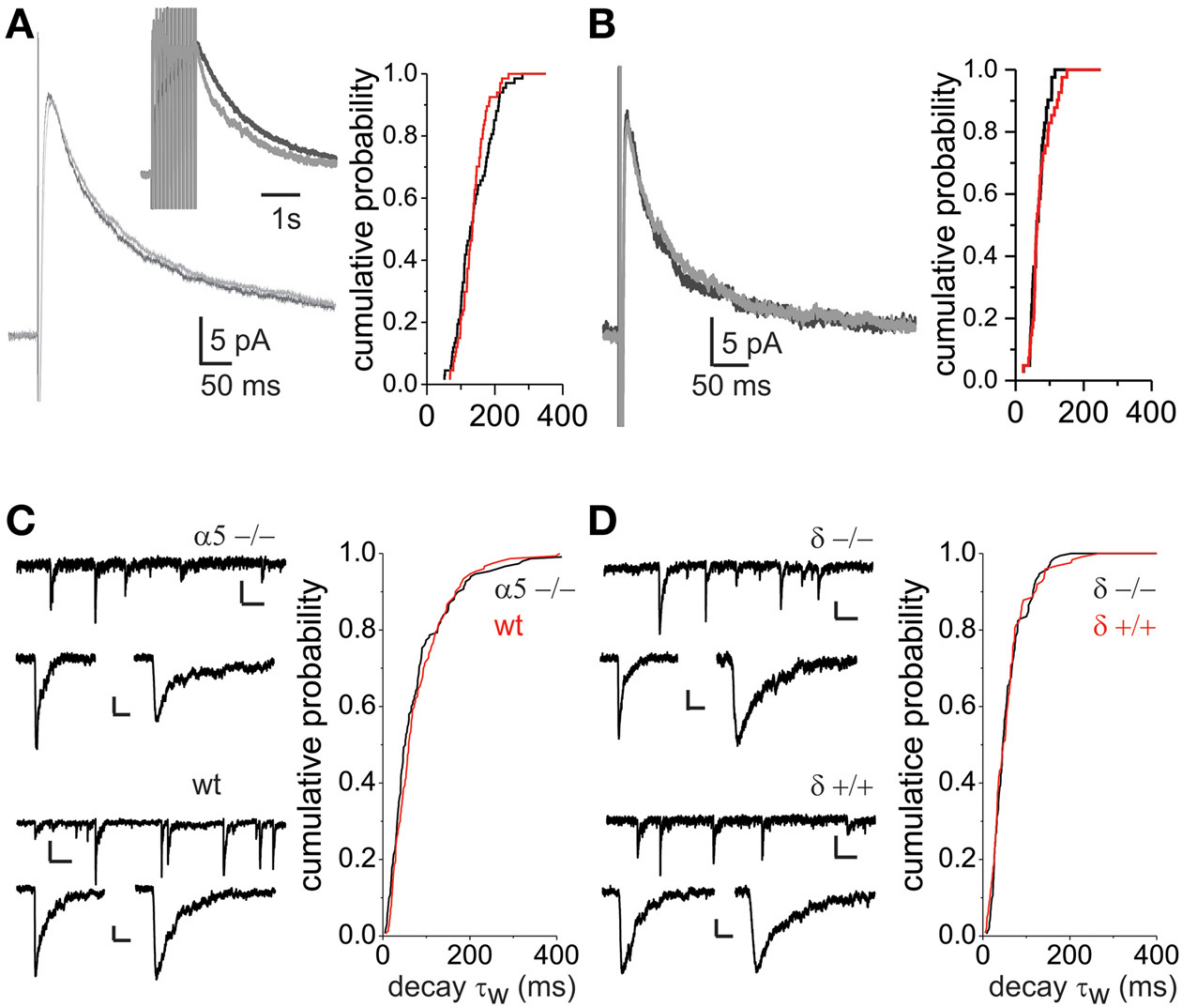
**Slow GABA_A_ IPSC decay kinetics in lamina II dorsal horn neurons are not explained by spillover or extrasynaptic receptor activation**. **(A)** Application of the GABA uptake blocker SKF89976A (SKF) does not affect the decay time of single GABA_A_ eIPSCs in GFP+ neurons. Traces on the left show average eIPSCs (0 mV holding potential) before (light gray) and during SKF application (black) recorded from a GFP+ neuron. On the right, the cumulative probability plots of eIPSC decay τ_w_'s from 5 GFP+ cells are shown (*p* > 0.05). The inset illustrates IPSCs evoked by a train of 12 stimuli at 20 Hz in the same cell (as in left) and shows a prolonged response after SKF application, indicating that block of uptake, in this case, revealed accumulation of GABA (the traces shown are normalized to the peak value). SKF prolonged the IPSC decay half-width after a train stimulus from 0.54 ± 0.08 to 0.65 ± 0.07 s (*n* = 5 GFP+ cells, *p* < 0.05). **(B)** Application of SKF does not affect the decay time of GABA_A_ eIPSCs in GFP− neurons. Traces on the left show average eIPSCs before (light gray) and during SKF application (black) recorded from a GFP− neuron. On the right, the cumulative probability plots of eIPSC decay τ_w_'s from 4 GFP− cells are shown (*p* > 0.05). **(C)** Traces of mIPSC recordings (scale bars: 10 pA, 0.5 s) and examples of single mIPSCs (scale bars: 10 pA, 50 ms) recorded from α5−/− (upper) and wild type (lower) mice. The cumulative probability plot shows identical distribution of decay τ_w_ between knock-out (6 cells) and control mice (6 cells). **(D)** Traces of mIPSC recordings (scale bars: 10 pA, 0.5 s) and examples of single mIPSCs (scale bars: 10 pA, 50 ms) recorded from δ −/− (upper) and δ +/+ (lower) mice. The distribution of decay time constants is similar in δ −/− (6 cells) and δ+/+ (5 cells) as shown by the cumulative probability plot.

Although transmitter released by single vesicles is not always sufficient to diffuse to activate extrasynaptic receptors or spillover (Isaacson et al., 1993; Overstreet and Westbrook, 2003), it might occur in certain circumstances because of the presence of extrasynaptic high affinity receptors, as was shown for certain glutamatergic synapses (Diamond, 2001). To directly examine if diffusion of GABA and activation of extrasynaptic receptors could be responsible for the slow decay component of GABA_A_ IPSCs, we looked at the effect of GABA uptake block. Evoked IPSCs were recorded in EGFP+ interneurons before and during the application of the GABA transporter blocker SKF89976A. Bath application of 100 μM SKF89976A did not significantly affect the decay time constant (τ_w_, 143 ± 6.9 vs. 137.3 ± 4.9; Figure 5A) in EGFP+ interneurons. Similarly, in EGFP− interneurons, SKF89976A did not have a significant effect on the decay time constant (69.6 ± 5.3 vs.74.5 ± 5.1; Figure 5B). On the other hand SKF89976A caused significant prolongation of the currents recorded after a 20 Hz train stimulus (Figure 5A). These results suggest that under our experimental conditions, diffusion and spillover of GABA does not contribute in the shaping of the slow decay kinetics observed in the eIPSCs of the dorsal horn.

### mIPSc decay kinetics are not altered in α5−/− and δ−/− mice

In additional experiments, we investigated the existence of slow decaying GABA_A_ mIPSCs in mice lacking the α5 or δ subunits. GABA_A_ receptors containing the α5 or δ subunits are high affinity receptors and are thought to be located extrasynaptically, mediating tonic inhibition (Mody and Pearce, 2004; Farrant and Nusser, 2005). We postulated that if the slowly decaying component of the synaptic current is due to activation of extrasynaptic receptors it might be mediated by either α5 or δ containing subunits. Figure 5C shows mIPSC recordings from α5−/− mice. Both mIPSCs with faster and prolonged decaying kinetics were observed. As shown in the cumulative probability plot the distribution of decay time constants in α5−/− mice did not differ from that of age-matched wild type mice. Similarly, slowly decaying mIPSCs were also observed in δ −/− mice (Figure 5D). As with the α5−/−, the decay time constant distribution in δ −/− was not significantly different from that of wild type littermates (δ +/+), confirming that GABA spillover and extrasynaptic receptor activation are not responsible for the slow decays.

### Heterogeneity of decay kinetics is replicated in experiments with rapid GABA application to spinal lamina II outside-out patches

**Figure 6:**
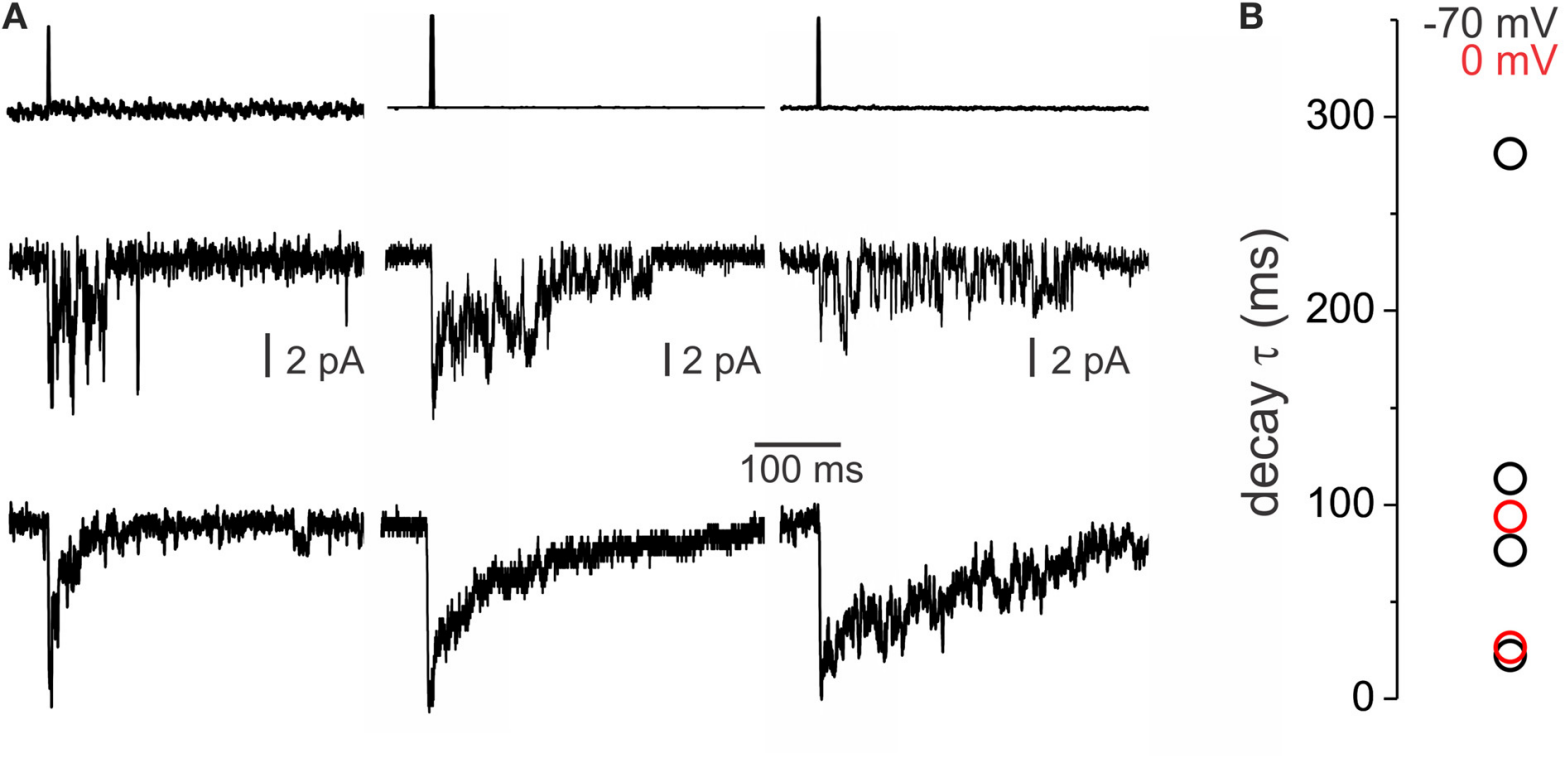
**Heterogeneity of decay kinetics of transient GABA_A_ currents in outside-out patches. (A)** Current traces showing responses to rapid application (1 ms pulse) of 1 mM GABA of varying decay times in different membrane patches. On top, the average of the liquid junction current recording is shown. In the middle, traces from a single application are shown. Average traces of multiple applications on the same membrane patches are shown at the bottom. **(B)** Graph showing the decay τ of individual excised patches. In black are shown outside-out patches recorded with high Cl− pipette solution and a holding of −70 mV while in red are recordings from excised patches made with low Cl− pipettes at 0 mV holding potential.

The above results indicate that intrinsic receptor properties and not extrinsic factors, like neurotransmitter time course and diffusion, shape the decay kinetics of GABA_A_ IPSCs. We sought to confirm this using conditions of controlled GABA application. Outside out membrane patches were isolated from lamina II neurons in mouse slices and exposed to brief pulses (1 ms) of GABA (1 mM) using a piezoelectric-controlled rapid application system. GABA induced the opening of several channels on the membrane patches (Figure 6A). Average traces for each patch were obtained from multiple brief pulse applications and the decay time constant was calculated by fitting a monoexponential decay function. Outside-out membrane patches displayed variable (Figure 6B) decay time constants ranging from 22.3 to 281.1 ms (*n* = 6), which corresponds to the range of decays we observed for mIPSCs. These data indicate that the intrinsic properties of GABA_A_ channels in lamina II interneurons are sufficient to explain the diversity of IPSC decay kinetics, consistent with the above findings that the range of decays observed are not due to differences in transmitter release or GABA reuptake mechanisms.

### Simulations reveal that dendritic filtering is not sufficient to explain the variability in decay kinetics, however diverse subunit composition could yield the observed variability

**Figure 7:**
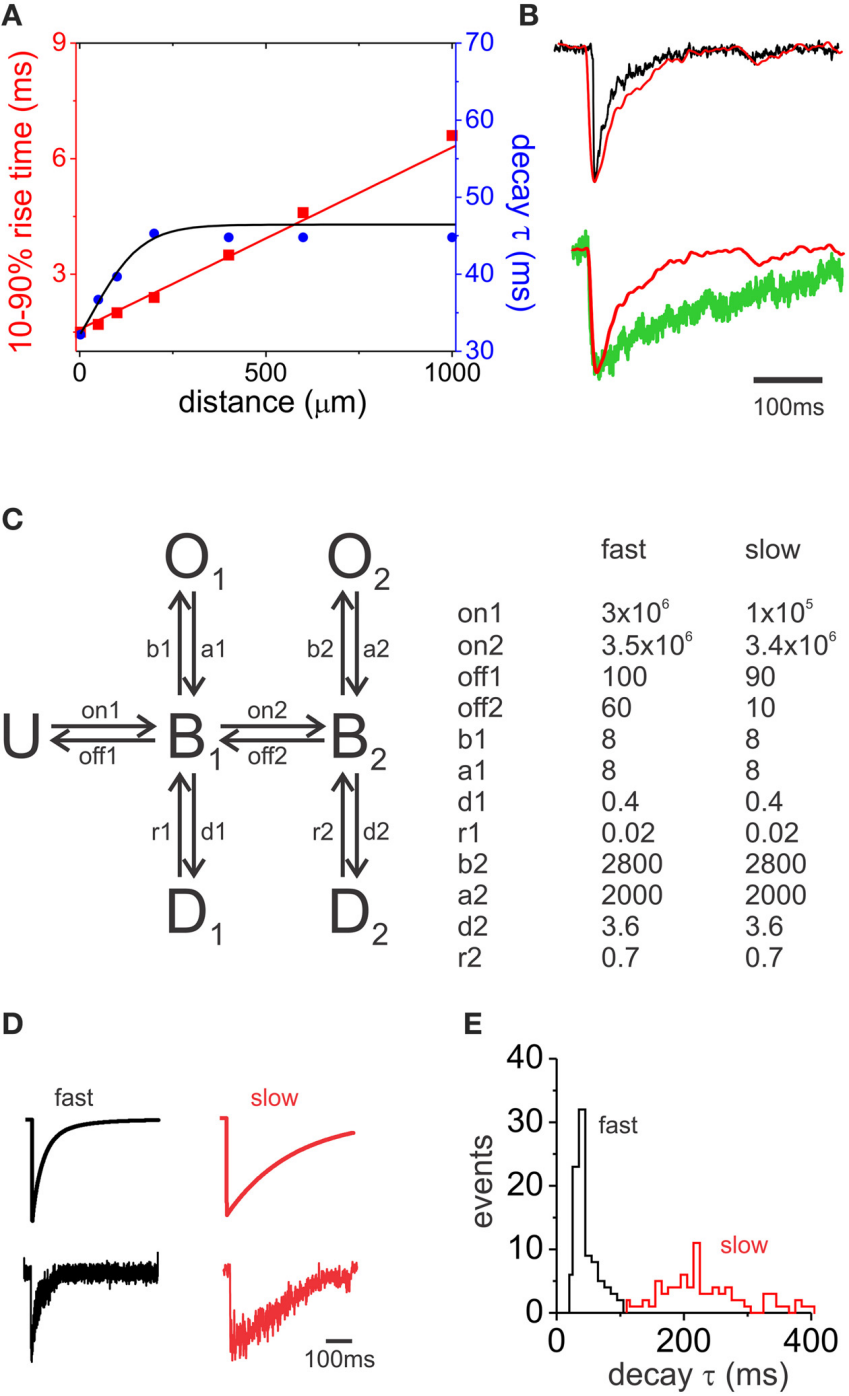
**Simulation of mIPSC decay kinetics**. **(A)** The graph illustrates the changes in 10–90% rise time and decay τ in relation to the distance of the synapse from the soma in a model cell. A recorded fast mIPSC was used as a synaptic conductance. **(B)** Top, superimposed synaptic currents measured at the soma for synapses located at 0 μm (black) and 1000 μm (red) away from the soma. Traces were aligned at the peak. Bottom, superimposed traces from a synaptic current originating 1000 μm away from the soma of the model cell (red) and a slow decaying recorded mIPSC (green). **(C)** Schematic representation of the six-state Markov model of GABA receptor activation used (left). U, unbound; B, bound; O, open,; and D, desensitized states. A list of rate constants used to simulate fast and slow decay GABA currents is shown on the right. on1 and on2 binding rate constants are in M^−1^s^−1^ and the rest rate constant in s^−1^. **(D)** Top, computationally generated macroscopic current simulations for fast and slow decay parameters. Bottom, examples of simulated GABA_A_ mIPSCs resulting from the summation of stochastic opening of 20 single channels. **(E)** Population histogram showing the distribution of decay τ for fast (black) and slow (red) simulated mIPSCs.

The SDH consists of a variety of interneurons with different morphologies. Soma size and dendritic length can differ considerably between excitatory and inhibitory inteneurons (Yasaka et al., 2007). These morphological differences could influence the dendritic filtering of synaptic events. To identify how much of the decay variability could be attributed to dendritic filtering we used simulation experiments on a simple model neuron consisting of a soma and a single dendrite. We used a recorded event and fed it as a synaptic conductance at different distances on the dendrite. Using this approach we were able to yield filtered rise times that compared to those in our recorded mIPSCs (Figure 7A). Filtering also slowed the decay kinetics, however the resulting variability is only a fraction of the recorded variability. This indicates that dendritic filtering would influence decay kinetics to a limited extent and cannot explain the full variability of mIPSC kinetics in our experiments (Figure 7B).

Subunit composition of GABA_A_ receptors can be a major factor in determining IPSC decay kinetics. A prominent example of cell type-specific and subunit-dependent differences in kinetic properties has been shown in the thalamus (Browne et al., 2001; Schofield and Huguenard, 2007). Inhibitory neurons from the nucleus reticularis display IPSCs with characteristic slow decay kinetics, while relay neurons in the ventrobasal area display fast decaying IPSCs, a difference attributable to GABA_A_ receptor affinity (Browne et al., 2001; Schofield and Huguenard, 2007). This raises the question of whether differences in the binding characteristics that result from distinct subunit composition are sufficient to explain the wide range of the mIPSC decay kinetics observed in spinal lamina II neurons. To test this, we used a six-state Markov model of channel gating to simulate mIPSC decays. This model (Figure 7C) was structurally similar to those previously described (Jones and Westbrook, 1995; Schofield and Huguenard, 2007). It included two binding states (mono-liganded B1 and bi-liganded B2), two open and two desensitized states. We first used rate constants similar to ones previously reported (Schofield and Huguenard, 2007) and modified them to obtain monoexponential macroscopic currents with a decay time constant equivalent to the fast decay component of our eIPSCs (average τ_1_ from EGFP+ and EGFP− eIPSCs; Table 1, high Cl^−^ recordings). We then reused the same parameters, but modified only GABA binding and unbinding rate constants to generate macroscopic currents with slow decay kinetics comparable to that of our eIPSCs (average τ_2_ from the eIPSCs in the two interneuron populations). Using these parameters, 100 “fast” and 100 “slow” mIPSCs were simulated each from stochastic opening of 20 single channels (Figure 7D). Figure 7E shows the distribution of decay time constants of the simulated mIPSCs, yielding a similar diversity of kinetics to that of mIPSCs recorded in lamina II interneurons. These results indicate that a difference in intrinsic parameters, such as binding and unbinding rates, is sufficient to replicate the heterogeneity of mIPSC observed among lamina II interneurons as was observed in the thalamus (Schofield and Huguenard, 2007). While not definitive proof, it is consistent with a differential subunit composition at different GABA_A_ synapses.

### Distinct α subunit composition explains the differential decay kinetics of GABA_A_ IPSCs

To test whether differences in α subunit composition of GABA_A_ receptors could explain the heterogeneity of IPSC decay kinetics, we exploited three lines of knock-in mice with altered α-subunit pharmacology. In these knock-in mice a histidine residue is substituted by an arginine at the benzodiazepine binding site of the α1 [α1(H101R)], α2 [α2(H101R)] or α3 [α3(H126R)] subunits (Rudolph et al., 1999; Low et al., 2000). The mutated subunits form functional GABA_A_ receptors (Benson et al., 1998) but are insensitive to diazepam modulation. On the other hand, Ro 15-4513 which acts as a partial inverse agonist on wild type GABA_A_ receptors, acts as an agonist on mutated benzodiazepine binding site. Thus, it selectively potentiates responses of GABA_A_ receptors that contain the point mutated α-subunits (Benson et al., 1998). This provided the missing pharmacological tool to test for the contribution of each of these subunits to the different kinetic components of GABA_A_ IPSCs.

**Figure 8:**
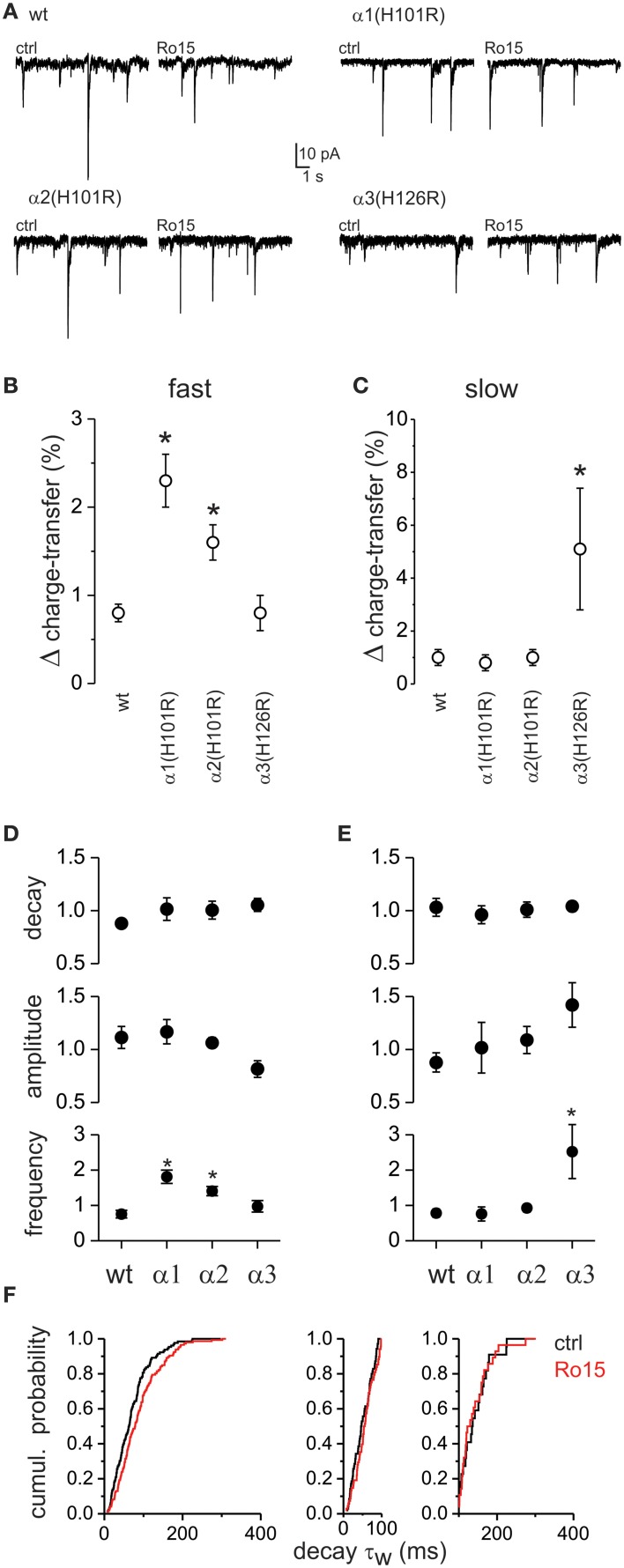
**Differential role of α-subunits in shaping GABA_A_ IPSC decay kinetics**. Effect of the partial benzodiazepine inverse agonist Ro15-4513 on fast and slow mIPSCs in control (wt), α1(H101R), α2(H101R), and α3(H126R) knock-in mice. **(A)** Example traces of mIPSC recordings before (ctrl) and during Ro 15-4513 in the different mouse lines. **(B)** The relative change in total mIPSC charge transfer (Δ charge-transfer) mediated by fast decay mIPSCs after the application of Ro15-4513. **(C)** The relative change in the Δ charge-transfer mediated by slow decay mIPSCs after the application of Ro15-4513. **(D)** The relative changes in mIPSC decay τ, amplitude and frequency for fast decaying mIPSCs after the application of Ro15-4513. **(E)** The relative changes in mIPSC decay τ, amplitude and frequency for slow decaying mIPSCs after the application of Ro15-4513. Asterisks denote significant difference between cells taken from wild type and knock-in mice (ANOVA with *post-hoc* Tukey test, *p* < 0.05, *n* = 9, 6, 6, and 6 respectively). **(F)** The cumulative probability plot of mIPSC decay τ_w_ for α3(H126R) knock-in mice (left) is shown before (ctrl) and during Ro 15-4513. Application of Ro 15-4513, shifted the decay τ distribution to the right, as expected by the increase in frequency of the slowly decaying mIPSCs subpopulation. Indeed, the cumulative probability plots for the fast (middle) and slow (right) subpopulations of the α3(H126R) mIPSC decay τ_w_ show no differences between ctrl and Ro15 in their distribution. All plots consist of pooled data from six α3(H126R) neurons. The same number of consecutively occurring mIPSCs from each cell was used.

In spinal slices taken form wild type and each of these knock-in mice, we recorded mIPSCs before (CTRL) and during the application of 1 μM Ro 15-4513 (Figure 8A). Amplitude, decay τ_w_ and frequency of the mIPSCs in CTRL did not differ between wild type and knock-in mice (not shown). To investigate decay-kinetic specific differences in the effect of Ro 15-4513 we categorized mIPSCs as fast, if their decay τ_w_ was <100 ms or as slow for mIPSCs with a τ_w_ > 100 ms. Benzodiazepines can modulate the amplitude, the decay time kinetics as well as the frequency of mIPSCs (in cases where it unmasks perisynaptic receptors for example, Chery and De Koninck, 1999). Hence, to measure the overall effect of Ro 15-4513, for each category of mIPSCs (fast and slow), we calculated the charge transfer for each mIPSC and then the sum of charge transfer for all mIPSCs in each category occurring over a period 10 min of CTRL and Ro 15-4513 conditions. We expressed the result as the ratio of Ro15-4513 to CTRL (Δ charge).

Comparison of Ro 15-4513 effect in the four groups of mice revealed a significant potentiation (*p* < 0.05) of the relative Δ charge contributed by fast mIPSCs in α1(H101R) and α2(H101R) mice, but not in α3(H126R) mice (*p* > 0.05) when compared with wt mice Figure 8B). In contrast, Ro 15-4513 significantly potentiated (*p* < 0.05) the relative Δ charge contributed by slow mIPSCs only in α3(H126R) mice (Figure 8C). The Ro 15-4513 effects on Δ charge for both slow and fast mIPSCs was mostly due to changes in event frequency (Figures 8D–F). These results indicate that GABA_A_ receptors containing α1 and α2 subunits are majorly responsible for shaping fast decaying mIPSCs while α3 underlies slow decay mIPSCs.

## Discussion

Here we demonstrate that GABA_A_ transmission is distinct in lamina II interneuron subpopulations as shown by the slower kinetics of evoked GABA_A_ IPSCs in inhibitory interneurons. Detailed analysis of mIPSC decay times showed that although both EGFP+ and EGFP− interneurons consist of mIPSC populations with similar decay kinetics, the relative distribution of faster and slower decay kinetics is distinct in the two populations. While in EGFP− interneurons are dominated by faster decaying mIPSCs, in EGFP+ interneurons slow decaying mIPSCs were more frequent on a relative scale. Furthermore, our data indicate that these differences reflect differential distribution of GABA_A_ receptor subunits.

Several lines of evidence suggest that the slower decaying mIPSCs are not the result of electrotonic filtering. The 10–90% rise time and decay time constants of mIPSCs do not correlate. When mIPSCs are subdivided to populations of faster, medium and slower decaying events each subpopulation shows similarly distributed rise times for each interneuron subtype. In addition, the existence of mIPSCs with mixed decay kinetics suggests that both components arise from the release of a single vesicle and thus the same electrotonic distance. Finally, in experiments from isolated membrane patches and rapid drug application a wide range in decay kinetics is observed. Thus, it is improbable that the observed variability in IPSC decay kinetics is due to dendritic filtering. However, we also observed slower mIPSC rise times in EGFP+ compared to EGFP− interneurons. This could be due to differences in electrotonic filtering in the two interneuron populations as a consequence of differences in morphology (Yasaka et al., 2007). However, other factors could also influence rise time, including differences in synaptic geometry or distinct subunit composition. Recent work in the rat dorsal horn using GABA and glycine uncaging suggests that the distribution of inhibitory neurotransmitter receptors in islet cells is mainly on the soma and proximal dendrites (Kato et al., 2007). In the same cells, electrotonic filtering of transients caused by glutamate uncaging only modestly affected decay time constants, providing evidence of electrically compact cells (Kato et al., 2007). Analogous results were provided by Chery and De Koninck (1999) for lamina I neurons where similar rise and decay kinetics were observed for both proximally and distally evoked IPSCs. In agreement with this the glycinergic eIPSC decay time constants in our experiments, as well as amplitudes, were equivalent in the two populations. Nonetheless, filtering could alter mIPSCs properties differently in EGFP− and EGFP+ interneurons. On the other hand, the similar rise time distribution for different decay kinetic populations, indicate that such filtering would affect the whole range of mIPSCs uniformly, thus it is unlikely it contributes to the differences in the mIPSC variability we observed between the two interneuron populations.

Slowly decaying GABA_A_ IPSCs have been observed before in several CNS areas. In the spinal ventral horn, Renshaw cells have significantly slower GABA_A_ IPSC decay kinetics than non-Renshaw cells which correlates with expression α3/α5-containing GABA_A_ receptors in these neurons (Geiman et al., 2002; Gonzalez-Forero and Alvarez, 2005). In the thalamus, GABAergic neurons in the reticular nucleus display slow decay IPSCs that are thought to arise from the expression of α3 subunits that confer lower receptor affinity for GABA (Browne et al., 2001; Schofield and Huguenard, 2007). Slow GABA_A_ IPSCs in hippocampal pyramidal neurons (Pearce, 1993), on the other hand, arise from low concentration and long lasting GABA transients that are evoked by neurogliaform cells (Karayannis et al., 2010) which also involve the activation of α5 containing GABA_A_ receptors (Zarnowska et al., 2009). Moreover, in the cerebellum, granule cells receive both fast and slow decay GABA_A_ IPSCs from the same type interneuron, the Golgi cell. While fast transients arise from activation of synaptic α1 containing receptors, slow transients involve spillover and the activation of high affinity α6 containing receptors (Rossi and Hamann, 1998). Neocortical low threshold-spiking (LTS) interneurons display slower IPSC decay kinetics than fast spiking (FS) interneurons, which correlates with the presence of α1 subunit in FS but lack of this subunit in LTS interneurons (Bacci et al., 2003). Finally, in pyramidal cortical neurons, similar to their hippocampal counterparts, low-concentration, slow GABA_A_ transients from neuroglia yield IPSCs with slow decay kinetics (Szabadics et al., 2007). These paradigms show that a multitude of mechanisms are involved in creating GABA_A_ IPSC kinetic diversity, including subunit composition but also variable synaptic arrangements yielding a wide variety of flavors of inhibition.

GABA clearance from the cleft by uptake transporters determines the duration of the neurotransmitter availability, its concentration and diffusion out of the cleft. All of these parameters also shape the decay kinetics of GABA_A_ IPSCs (Isaacson et al., 1993; Nusser et al., 2001; Overstreet and Westbrook, 2003). Our experiments indicate that the neurotransmitter time course in the synapse plays little role in shaping the decay time course of IPSCs in spinal lamina II, since pharmacological blockade of GABA uptake did not affect the kinetics of single eIPSCs. The presence of slow decay mIPSCs also argues against spillover. The occurrence of slow mIPSCs in knockouts of the high affinity α5 and δ subunits is consistent with these conclusions. Finally, we confirmed the role of intrinsic receptor properties as sufficient to explain the heterogeneity of decay kinetics with outside out membrane patches under controlled GABA applications.

The subunit composition of GABA_A_ receptors is a major determinant of their pharmacological and biophysical properties (Macdonald and Olsen, 1994; Gingrich et al., 1995; Olsen and Sieghart, 2009). Immunocytochemical and *in situ* hybridization studies in the spinal cord have shown the expression of several subunits, including α1, α2, α3, and α5, the β 2 and β 3, and the γ2 subunit (Persohn et al., 1991; Wisden et al., 1991; Ma et al., 1993; Bohlhalter et al., 1996; Todd et al., 1996). Furthermore, in recent functional studies, the spinal α2 subunit and, to a lesser extent, α3 and α5 have been implicated in the analgesic effect of diazepam in neuropathic and inflammatory pain (Knabl et al., 2008). The distinct effects of Ro 15-4513 on the fast and slow decay mIPSCs in the three knock-in mice establishes the GABA_A_ receptor composition as the main factor contributing to the heterogeneity in IPSC decay kinetics in spinal lamina II. Consistent with our findings, fast decay IPSCs have been previously associated with the α1 subunit in other brain areas (Browne et al., 2001; Vicini et al., 2001; Bacci et al., 2003). Also consistent with our findings, α3 subunits have been shown to underlie slow decay IPSCs in the ventrobasal thalamus (Browne et al., 2001; Schofield and Huguenard, 2007). In addition to these observations, further variability in GABA_A_ receptor kinetics may result from receptor phosphorylation (Nusser et al., 1999) or from modulation by endogenous neurosteroids (Keller et al., 2004; Poisbeau et al., 2005). In the SDH a regional variability of GABA_A_ decay kinetics was shown to be a result of local differences in steroid synthesis (Inquimbert et al., 2008). Variation in menstrual cycle hormones might also contribute to an additional variation in kinetics (Poisbeau et al., 2014).

The heterogeneity in kinetics between different cell types and even within the same neuron at different synapses, implies multiple functionality of the GABA_A_ synapse. Indeed, distinct GABA_A_ receptor kinetics can differentially affect information processing. While fast inhibitory currents can modulate the threshold of input-output transfer (Crowley et al., 2009), slow and tonic inhibition can influence both the slope (gain) and threshold of input-output transfer (Mitchell and Silver, 2003; Prescott and De Koninck, 2003; Crowley et al., 2009). Consequently, activation of a subset of synapses with a specific subunit composition can result in distinct processing and information flow. This raises the question of whether synapses with certain subunit composition and kinetics (fast or slow) receive specialized input. At present our understanding of the information processing in the SDH is too poor to conclude on this issue because several key pieces of information on the structural and functional organization in this area are still missing and further investigations are warranted.

Taken together the present results provide evidence that distinct subunit composition underlies the heterogeneity in time course of inhibition in the dorsal horn of the spinal cord. This finding may have important implications for pain control and drug design, as they may contribute to our understanding of the net impact drugs may have at the network level. In recent studies, molecular dissection of the analgesic effect of diazepam has shown that it is mainly mediated by the α2 and, to a lesser extent, α3 subunits at the spinal level (Knabl et al., 2008). These results open new avenues for specific pharmacological targeting of GABA_A_ receptors subtypes for pain treatment. Yet, the α3 subunit did not appear to contribute to benzodiazepine-mediated analgesia to the same degree in the different pain tests performed (Knabl et al., 2008). In this context, it is interesting to note that, from our results, the α3 subunit appears to dominate inhibition of inhibitory interneurons. Potentiation of this subunit on these interneurons may thus cause significant disinhibition. Because distinct elements of the dorsal horn circuitry may be affected differentially in each chronic pain condition and in each sensory response, the net effect of modulating the α3 subunit may differ. In certain cases, for example, the “pro-algesic” effect of disinhibition may counteract any concomitant analgesic action resulting from inhibition of dorsal horn excitatory circuits. Thus, our results suggest that selective targeting of the α2 subunit may yield a more broadly effective analgesic.

## Author contributions

Charalampos Labrakakis and Yves De Koninck conceived the study. Charalampos Labrakakis designed, performed and analyzed the experiments. All authors contributed to the interpretation of data and preparation of the final manuscript.

### Conflict of interest statement

The authors declare that the research was conducted in the absence of any commercial or financial relationships that could be construed as a potential conflict of interest.
